# Phase II study of weekly oxaliplatin and 24-h infusion of high-dose 5-fluorouracil and folinic acid in the treatment of advanced gastric cancer

**DOI:** 10.1038/sj.bjc.6601985

**Published:** 2004-06-29

**Authors:** Y Chao, K H Yeh, C J Chang, L T Chen, T Y Chao, M F Wu, C S Chang, J Y Chang, C Y Chung, W Y Kao, R K Hsieh, A L Cheng

**Affiliations:** 1 Taipei Veterans General Hospital, Taipei, Taiwan; 2 National Taiwan University Hospital, Taipei, Taiwan; 3 Far Eastern Memorial Hospital, Taipei, Taiwan; 4 Tri-Service General Hospital, Taipei, Taiwan; 5 Chung Shan Medical and Dental College Hospital, Taipei, Taiwan; 6 Changhua Christian Hospital, Taipei, Taiwan; 7 Mackay Memorial Hospital, Taipei, Taiwan; 8 National Health Research Institutes, Taipei, Taiwan

**Keywords:** oxaliplatin, high-dose 5-fluorouracil, folinic acid, advanced gastric cancer

## Abstract

To investigate the efficacy and safety of combining weekly oxaliplatin with weekly 24-h infusion of high-dose 5-fluorouracil (5-FU) and folinic acid (FA) in treatment of patients with advanced gastric cancer. Patients with histologically confirmed, locally advanced or recurrent/metastatic gastric cancer were studied. Oxaliplatin 65 mg m^−2^ 2-h intravenous infusion, and 5-FU 2600 mg m^−2^ plus FA 300 mg m^−2^ 24-h intravenous infusion, were given on days 1 and 8, repeated every 3 weeks. Between January 2001 through January 2002, 55 patients were enrolled. The median age was 64 years (range: 22–75). In all, 52 patients (94.5%) had recurrent or metastatic disease and three patients had locally advanced disease. Among 50 patients evaluable for tumour response, 28 patients achieved partial response, with an overall response rate of 56% (95% confidence interval (CI): 41.8–70.3%). All 55 patients were evaluated for survival and toxicities. Median time to progression and overall survival were 5.2 and 10.0 months, respectively, during median follow-up time of 24.0 months. Major grades 3–4 toxicities were neutropenia in 23 cycles (7.1%) and thrombocytopenia in 16 cycles (5.0%). Treatment was discontinued for treatment-related toxicities in nine patients (16.4%), of whom eight were due to oxaliplatin-related neurotoxicity. One patient (1.8%) died of neutropenic sepsis. This oxaliplatin-containing regimen is effective in the treatment of advanced gastric cancer. Except for neurotoxicity that often develops after prolonged use of oxaliplatin, the regimen is well tolerated.

Gastric cancer is the second leading cause of cancer death worldwide (Ho, 1988; Roder, 2002). The prognosis is generally poor, with overall 5-year survival ranged from 5 to 15%. Most patients present with advanced or metastatic diseases, for which curative resection is not possible. Chemotherapy is used primarily for palliation of symptoms (Findlay and Cunningham, 1993; Wils, 1996; De Vivo *et al*, 2000). In the past three decades, a variety of chemotherapy regimens were developed for the treatment of advanced gastric cancer (MacDonald *et al*, 1980; Preusser *et al*, 1989; Wilke *et al*, 1990; Wils *et al*, 1991; Kim *et al*, 1993; Cocconi *et al*, 1994; Zaniboni *et al*, 1995; Ychou *et al*, 1996; Cheng *et al*, 1998). All these regimens had variable degrees of success in phase II trials; however, results of the subsequent phase III trials often failed to confirm the relatively high response rates of earlier reports (Kelsen *et al*, 1992; Kim *et al*, 1993; Cocconi *et al*, 1994; Webb *et al*, 1997; Vanhoefer *et al*, 2000). The survival benefit was limited with overall survival consistently below 10 months, and substantial treatment-related toxicities were observed in most regimens (Waters *et al*, 1999; Ajani *et al*, 2003; Ohtsu *et al*, 2003). Further, second-line chemotherapy is hardly effective in most trials, indicating the rapid emergence of drug resistance of gastric cancer cells to currently available anticancer drugs (Findlay and Cunningham, 1993; Wils, 1996; De Vivo *et al*, 2000). New treatments with better therapeutic index or novel agents with lesser cross-resistance are needed.

Oxaliplatin is a third-generation platinum compound that has a wide range of antitumour activities (Machover *et al*, 1996; Monnet *et al*, 1998; de Gramont *et al*, 2000; Misset *et al*, 2001). Compared with cisplatin, oxaliplatin appears to have a better safety profile; and the cross-resistance to cisplatin is minimal (Kollmannsberger *et al*, 2002). Oxaliplatin has higher anticancer activity than cisplatin in some preclinical experiments (Di Francesco *et al*, 2002). Synergism between oxaliplatin and 5-fluorouracil (5-FU) has been demonstrated *in vitro* (Pendyala and Creaven, 1993; Hsu *et al*, 2000), and *in vivo* (Andre *et al*, 1999). Combination of oxaliplatin and 5-FU has proven effective as first- or second-line treatment for advanced colorectal cancer (Machover *et al*, 1996; de Gramont *et al*, 2000). Preliminary results of several recent studies have suggested that various combinations of oxaliplatin and 5-FU may be as effective in gastric cancer as in colorectal cancer.

We have previously demonstrated that weekly 24-h infusion of high-dose 5-FU and folinic acid (FA), the HDFL regimen originally described by Ardalan *et al* (1991), appears to be particularly useful in gastric cancer (Yeh and Cheng, 1994; Hsu *et al*, 1997; Yeh and Cheng, 1998). We have also provided evidence that prolonged exposure of gastric cancer cells to low concentration 5-FU for 24 h enhances the inhibition of thymidylate synthase, and thereby increases the cytotoxicity of 5-FU (Yeh *et al*, 2000b). Further, HDFL regimen has repeatedly been demonstrated to cause minimal myelosuppression (Hsu *et al*, 1997; Yeh *et al*, 1997; Yeh and Cheng, 1998) and is therefore one of the ideal components for combination chemotherapy with other cytotoxic agents against gastric cancer. The possible mechanism responsible for the very low myelotoxicity of HDFL has recently been reported (Yeh *et al*, 2000a).

This study sought to investigate if combination of oxaliplatin and HDFL may be a regimen with better therapeutic index for patients with advanced gastric cancer.

## PATIENTS AND METHODS

### Patients

Eligibility criteria of the patients included (1) pathologically confirmed, locally advanced (nonresectable), recurrent or metastatic gastric cancer, (2) objectively measurable disease by imaging studies, (3) no prior chemotherapy except postoperative adjuvant chemotherapy that was received more than 12 months before entry into the study, (4) ECOG (Eastern Cooperative Oncology Group) performance status ⩽2, (5) age between 18 and 75 years, (6) adequate hepatic, renal, and bone marrow functions, and (7) serum triglyceride >70 mg dl^−1^. The low limit for serum triglyceride was set to avoid HDFL-related hyperammonaemic encephalopathy, which occurs in around 5% of Taiwanese patients (Yeh and Cheng, 1997).

Exclusion criteria included (1) pre-existing peripheral neuropathy, (2) pregnant, breastfeeding, or woman of child-bearing potential without adequate contraception, (3) concurrent or prior malignancy except curatively resected cervical carcinoma *in situ* or squamous cell carcinoma of skin, (4) central nervous system metastases, (5) active infection, and (6) concurrent treatments that interfered with study evaluation. This study was approved by the ethics committee of all participating institutes. Signed informed consent was obtained from all patients.

### Study design

This is a prospective, multicentre, phase II clinical trial.

### Chemotherapy protocol

On days 1 and 8 of each cycle of chemotherapy, oxaliplatin 65 mg m^−2^ was given as a 2-h intravenous infusion, then followed by 5-FU 2600 mg m^−2^ and FA 300 mg m^−2^, as a continuous 24-h intravenous infusion. Treatment was repeated every 21 days. Treatment was continued until disease progression or unacceptable toxicity developed. Patients with complete response received at least two more cycles of chemotherapy.

### Dose modification

Subsequent cycle of chemotherapy was withheld if peripheral blood neutrophils <1500 mm^−3^ or platelets <100 000 mm^−3^ was noted on the due day. If recovery of neutrophils and platelets took more than 3 weeks after the due day, the patient was removed from protocol treatment. 5-FU was decreased to 2000 mg m^−2^ for subsequent cycles if grade 4 thrombocytopenia or neutropenia developed in the precedent cycle of chemotherapy. Dose of 5-FU was further reduced to 1600 mg m^−2^ for subsequent cycles if grade 4 thrombocytopenia or neutropenia developed again after first level of dose reduction of 5-FU. Oxaliplatin was decreased to 50 mg m^−2^ if grade 4 thrombocytopenia or neutropenia developed after two levels of 5-FU dose reduction. If grade 3–4 nonhaematological toxicities or grade 2 hand–foot syndrome developed, 5-FU was decreased to 2000 mg m^−2^ for subsequent cycles. Dose of 5-FU was further decreased to 1600 mg m^−2^ for subsequent cycles if grade 3–4 nonhaematological toxicities or grade 2 hand–foot syndrome recurred after first levels of dose reduction of 5-FU. Oxaliplatin was decreased to 50 mg m^−2^ if grade 3–4 nonhaematological toxicities or grade 2 hand–foot syndrome developed again after two levels of 5-FU dose reduction. For grade 2–3 neurotoxicities, oxaliplatin was omitted until recovery of the neurotoxicities to grade 1 or less; and the dose of oxaliplatin was decreased to 50 mg m^−2^ for subsequent cycles. Oxaliplatin was discontinued if grade 2–3 neurotoxicities lasted for more than 3 weeks.

### Evaluation of efficacy and toxicities

Evaluations before chemotherapy included medical history taking, physical examination, complete blood count, blood chemistry, chest X-ray, computed tomography (CT) scan of abdomen, and gastroendoscopy. After starting protocol treatment, complete blood count was examined weekly and blood chemistry every 3 weeks. Patients' condition and treatment-related toxicities were evaluated weekly. Tumour size was measured by imaging studies every 6 weeks. Tumour response was evaluated according to the World Health Organization criteria. Toxicities were graded using the NCI-Common Toxicity Criteria (version 1).

### Statistical methods

The Simon two-stage design was used. The response rates of interest were *P*_0_=40% and *P*_1_=60%. If there were more than 12 responses in 29 patients in the first stage, the study would continue to 54 patients in the second stage. If there were more than 27 responses in 54 patients in the second stage, this treatment would be acceptable with *α* of 0.05 and *β* of 0.10.

Time to progression was defined as the duration from the date of starting protocol treatment to the date of documented disease progression or death by any cause. The overall survival was defined as the duration from the date of starting protocol treatment to the date of death. Kaplan–Meier method was used in all survival analyses.

## RESULTS

### Patients and treatment

Between January 2001 through January 2002, 55 patients were enrolled into the study. Major clinicopathologic features of the patients are listed in Table 1

**Table 1:** Clinicopathologic features of the patients

	**Patient number (%)**
Total patients	55
Sex: male/female	36/19
ECOG performance	
0	9 (16.4)
1	39 (70.9)
2	7 (12.7)
*Treatments for primary tumour*	
No prior therapy	31 (56.4)
Surgery only	18 (32.7)
Surgery + adjuvant chemotherapy	6 (10.9)
*Disease status*	
Locally advanced	3 (5.5)
Recurrence/metastasis	52 (94.6%)
*Disease sites*	
Liver	26 (47.3)
Lymph nodes	28 (50.9)
Peritoneum	15 (27.3)
Gastrointestinal tract	9 (16.4)
Bone	9 (16.4)
Lung	5 (9.1)
Others	11 (20)

. The median age of the patients was 64 years (range: 22–75). A total of 323 cycles (median: 6, range: 1–17) of chemotherapy were given. Median relative dose intensity was 95% (range: 67–100%) for oxaliplatin, 95% (range: 48–100%) for 5-FU, and 95% (range: 48–100%) for FA. As oxaliplatin has a cumulative toxicity, the median relative dose intensity of oxaliplatin was 100% (range: 70–100%) at cycle 3 and 88% (range: 67–100%) at cycle 6, respectively. In total, 75% of the patients received more than 80% of intended doses of oxaliplatin, 5-FU and FA.

### Efficacy

Five patients were not evaluable for response: four failed to return to the clinic for tumour measurements and one later found to have nonmeasurable tumours. Of the 50 patients evaluable for tumour response, there were 28 patients with partial remission (PR), four patients with stable disease (SD), and 18 patients with progressive disease (PD). The total tumour response rate was 56% (95% CI: 41.8–70.3%). The median time to tumour response was 3 months (range: 2.2–8.3 months). Five out of the six patients, who had previously received postoperative adjuvant chemotherapy which contains cisplatin and 5-FU, achieved PR. Two responders went on to receive curative surgical resection or radiotherapy for the residual tumours. Both patients were alive and tumour-free at 16 and 25 months after starting protocol chemotherapy, respectively.

**Figure 1:**
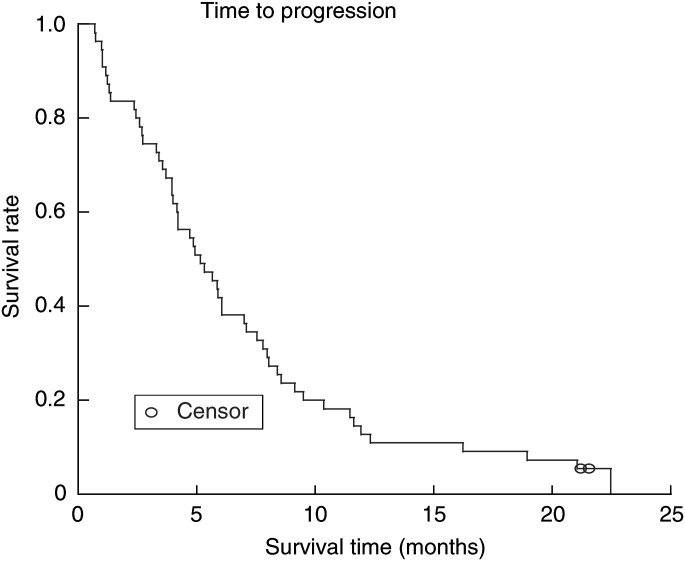
Time to progression of the 55 patients.

**Figure 2:**
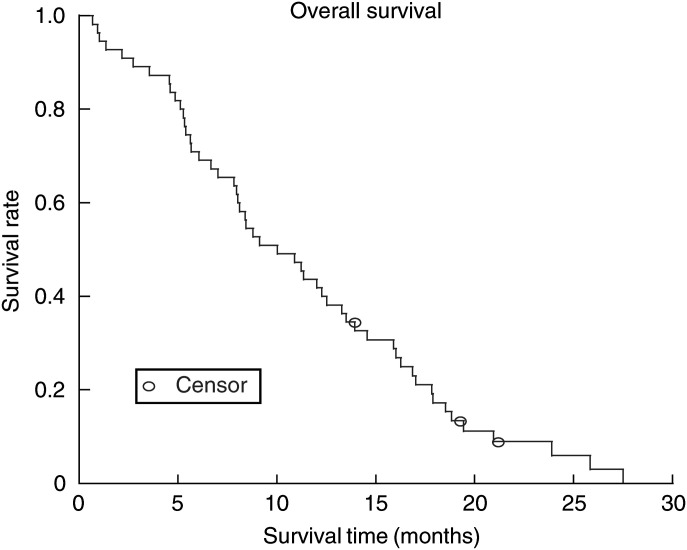
Overall survival of the 55 patients.

Median follow-up time of the whole group of 55 patients was 24.0 months as cut-off date for analysis on 25 July 2003. The median time to progression was 5.2 months (95% CI: 4.0–7.0 months) (Figure 1

). The median overall survival was 10.0 months (95% CI: 8.0–13.3 months) (Figure 2

).

### Toxicity

All 55 patients were evaluated for toxicities (Table 2

**Table 2:** Toxicity of the oxaliplatin-HDFL regimen

	**Patients (*n*=55)**		**Cycles (*n*=323)**	
	**Grade**	**Grade**	**Grade**	**Grade**
**Toxicity**	**1–2**	**3–4**	**1–2**	**3–4**
*Haematological*				
Neutropenia	72.7^a^	21.8	34.3^b^	7.1
Leukopenia	58.1	7.2	21.0	1.8
Thrombocytopenia	60.0	12.7	24.5	5.0
Febrile neutropenia	1.8	5.4	0.3	0.9
Anaemia	61.8	9.0	34.7	2.8
*Gastrointestinal*				
Nausea	81.8	10.9	38.4	2.5
Vomiting	87.2	12.7	31.6	2.2
Diarrhoea	69.1	12.7	22.0	2.4
Stomatitis	50.9	3.6	13.3	0.9
Anorexia	70.9	1.8	36.5	0.3
Weight loss	9.1	0	1.85	0
*Neuropathic*	72.7	12.7	46.8	2.2
*Others*				
Cardiac	0	1.8	0	0.3
Fever	27.3	1.8	7.1	0.3
Infection without neutropenia	16.4	0	4.3	0
Alopecia	21.8	0	16.4	0

a All numbers are percentage of the 55 patients.

b All numbers are percentage of the 323 cycles given.

). Nine patients (14.5%) discontinued treatment because of treatment-related toxicity. Eight out of these nine patients discontinued treatment because of oxaliplatin-related neurotoxicity. The median dose of oxaliplatin received by these eight patients was 910 mg m^−2^ (range: 715–1170 mg m^−2^). Another patient discontinued treatment due to heart failure, which was considered unrelated to chemotherapy.

## DISCUSSION

The results of this phase II study indicated that weekly oxaliplatin and 24-h infusion of high-dose 5-FU and FA is an effective combination chemotherapy for advanced gastric cancer. The overall response rate of 56% was within the range (30–70%) of previous major protocols such as FAMTX, ELF, EAP, ECF, and recent taxane-based (Bokemeyer *et al*, 1997; Kollmannsberger *et al*, 2000; Cascinu *et al*, 2001) or irinotecan-based regimens (Bleiberg, 1999; Ajani *et al*, 2002; Slater *et al*, 2002). Recently, three other studies, using different administrating schedules of oxaliplatin and 5-FU, have also demonstrated a good efficacy in gastric cancer (Louvet *et al*, 2002; Kim *et al*, 2003; Al-Batran *et al*, 2004). These phase II studies showed a response rates of 43–44.9% (Louvet *et al*, 2002; Al-Batran *et al*, 2004) and 26% (Kim *et al*, 2003) in the first- and second-line treatment, respectively. Oxaliplatin appears to be a useful adjunct to systemic chemotherapy against advanced gastric cancer. Currently, a randomised multicentre study (REAL-2) is underway with a two by two factorial design to compare the efficacy of capecitabine with 5-FU, and oxaliplatin with cisplatin in the ECF regimen, for patients with advanced oesophagogastric cancer. The study aims to enroll 1000 patients with the primary end point being 1-year survival. The interim analysis of the REAL-2 study showed good antitumour activity in favour of oxaliplatin and capecitabine with a response rate of 52% (95% CI: 34.4–68.1%) in an EOX (epirubicin, oxaliplatin, capecitabine) regimen (Sumpter *et al*, 2003).

In this study, 5-FU and FA was given by two weekly 24-h infusion (the HDFL regimen) every 3 weeks. The rationale of this scheduling for 5-FU was based on our previous observations which indicated that HDFL is in general a highly effective and very safe regimen for advanced gastric cancer (Hsu *et al*, 1997; Yeh and Cheng, 1998). The patients' compliance was excellent. To avoid calcite precipitation and catheter blockage (Ardalan *et al*, 1991, 1995), we decreased the dose of FA from 500 to 300 mg m^−2^, mixed the high-dose 5-FU and FA in the same infusion bag, and administered via an ambulatory infusion pump (Yeh and Cheng, 1994). This effective, nonmyelosuppressive, convenient outpatient-based administration of HDFL has been widely used at many institutions in Taiwan since 1994 (Yeh and Cheng, 1994), instead of much more complicated administration of LV5FU2 regimen (de Gramont regimen) in FOLFOX combination (de Gramont *et al*, 2000). A phase II study of high-dose 5-FU (2600 mg m^−2^) and FA (500 mg m^−2^), German AIO regimen, has also been reported as a salvage regimen in gastric cancer with partial remissions of 18% and stable disease of 41% (Vanhoefer *et al*, 1994). The decreased dose of FA (300 mg m^−2^) in our HDFL compared to German AIO regimen may also decrease the toxicity of grade 3/4 diarrhoea (Kohne *et al*, 2003). Further, results of our *in vitro* studies have implied that strict avoidance of bolus injection of 5-FU is the key to avoid myelosuppression (Yeh *et al*, 2000a). Although the best protocol of 5-FU remains to be explored (Johnson *et al*, 1991; Louvet *et al*, 1991), both clinical and laboratory data indicate that HDFL is one of the ideal components for combination chemotherapy with other cytotoxic agents against gastric cancer.

In this study, five out of six patients who had received prior adjuvant chemotherapy which contains cisplatin and 5-FU responded to the current protocol. A similar observation has recently been reported by Kim *et al* (2003). It appears that oxaliplatin has no significant cross-resistance with cisplatin in gastric cancer. Therefore, it is intriguing to examine if oxaliplatin is effective in second-line chemotherapy for gastric cancer patients recurrent from cisplatin-containing regimens.

Although survival is not a reliable end point for evaluation of efficacy in phase II trials, the median overall survival of 10.0 months in this study compared favourably to ECF (8.7 months), FAMTX (6.1 months), FAMTX (6.7 months), ELF (7.2 months), FUP (7.2 months), and FOLFOX 6 (8.6 months) (Webb *et al*, 1997; Vanhoefer *et al*, 2000; Louvet *et al*, 2002). It also compares favourably to prior studies conducted in our institutions on similar groups of patients (Hsu *et al*, 1997; Cheng *et al*, 1998; Chi *et al*, 1998).

Except for the oxaliplatin-related neurotoxicity (Cassidy and Misset, 2002), the current oxaliplatin-HDFL protocol is generally well tolerated. Neurotoxicity was also found to be the predominant dose-limiting toxicity in another oxaliplatin-containing regimen for gastric cancer (Louvet *et al*, 2002). Other major toxicities, including neutropenia, thrombocytopenia, stomatitis, and diarrhoea, were of the same range of severity as other major regimens such as FAMTX, ECF, FUP, ELF, and FOLFOX6 (Webb *et al*, 1997; Vanhoefer *et al*, 2000; Louvet *et al*, 2002). Owing to patients' selection in phase II studies, it may not be possible or relevant to compare the toxicity profiles of the present study with three other phase II studies, using different administrating schedules of oxaliplatin and 5-FU in gastric cancer (Louvet *et al*, 2002; Kim *et al*, 2003; Al-Batran *et al*, 2004). However, the haematological toxicity of the present study is still favourable and well tolerated with grade 3–4 neutropenia and febrile neutropenia of 7.1 and 0.9%, respectively, in a total of 323 cycles given. Further, the absence of grade-2 alopecia in this study compared favourably to other regimens.

We conclude that combination of weekly oxaliplatin and weekly 24-h infusion of 5-FU and FA is an active regimen with acceptable toxicities for the treatment of advanced gastric cancer.
